# Genotyping‐by‐sequencing of genome‐wide microsatellite loci reveals fine‐scale harvest composition in a coastal Atlantic salmon fishery

**DOI:** 10.1111/eva.12606

**Published:** 2018-03-11

**Authors:** Ian R. Bradbury, Brendan F. Wringe, Beth Watson, Ian Paterson, John Horne, Robert Beiko, Sarah J. Lehnert, Marie Clément, Eric C. Anderson, Nicholas W. Jeffery, Steven Duffy, Emma Sylvester, Martha Robertson, Paul Bentzen

**Affiliations:** ^1^ Science Branch Department of Fisheries and Oceans Canada St. John's NL Canada; ^2^ Department of Biology Dalhousie University Halifax NS Canada; ^3^ Faculty of Computer Science Dalhousie University Halifax NS Canada; ^4^ Center for Fisheries Ecosystems Research Fisheries and Marine Institute of Memorial University of Newfoundland St. John's NL Canada; ^5^ Labrador Institute of Memorial University of Newfoundland Happy Valley‐Goose Bay NL Canada; ^6^ Fisheries Ecology Division Southwest Fisheries Science Center National Marine Fisheries Service National Oceanic and Atmospheric Administration Santa Cruz CA USA

**Keywords:** genetic assignment, microsatellite, mixed‐stock analysis, next‐generation sequencing, *Salmo salar*

## Abstract

Individual assignment and genetic mixture analysis are commonly utilized in contemporary wildlife and fisheries management. Although microsatellite loci provide unparalleled numbers of alleles per locus, their use in assignment applications is increasingly limited. However, next‐generation sequencing, in conjunction with novel bioinformatic tools, allows large numbers of microsatellite loci to be simultaneously genotyped, presenting new opportunities for individual assignment and genetic mixture analysis. Here, we scanned the published Atlantic salmon genome to identify 706 microsatellite loci, from which we developed a final panel of 101 microsatellites distributed across the genome (average 3.4 loci per chromosome). Using samples from 35 Atlantic salmon populations (*n* = 1,485 individuals) from coastal Labrador, Canada, a region characterized by low levels of differentiation in this species, this panel identified 844 alleles (average of 8.4 alleles per locus). Simulation‐based evaluations of assignment and mixture identification accuracy revealed unprecedented resolution, clearly identifying 26 rivers or groups of rivers spanning 500 km of coastline. This baseline was used to examine the stock composition of 696 individuals harvested in the Labrador Atlantic salmon fishery and revealed that coastal fisheries largely targeted regional groups (<300 km). This work suggests that the development and application of large sequenced microsatellite panels presents great potential for stock resolution in Atlantic salmon and more broadly in other exploited anadromous and marine species.

## 1 INTRODUCTION

The maintenance of intraspecific diversity has been linked to both species and fishery persistence and stability (Hilborn, Quinn, Schindler, & Rogers, 2003; Schindler et al., 2010), and as such is central to successful wildlife and fisheries management (Funk, McKay, Hohenlohe, & Allendorf, 2012). In many instances, the management of intraspecific diversity relies on genetic and genomic descriptions of population structure, with individual assignment and genetic mixture analysis the mainstays of wildlife and fisheries management (Manel, Gaggiotti, & Waples, 2005). Both genetic‐based assignment and mixture analysis have been widely implemented in the management of a variety of taxa including mammals (Baker et al., 2010; Puckett & Eggert, 2016; Wasser et al., 2015), marine fishes and invertebrates (Benestan et al., 2015; Bradbury et al., 2011; Bradbury, Hamilton, Sheehan et al., 2016), and migratory birds (Ruegg et al., 2017). With the increasing availability of genetic and genomic resources for nonmodel species, opportunities exist for enhanced integration of assignment and mixture approaches into fisheries and wildlife management and for substantial improvements to current methods for resolving stock structure.

A variety of genomic tools exist for population resolution and assignment, but increasingly applications are favoring the use of single nucleotide polymorphisms (SNPs) over microsatellite loci (Guichoux et al., 2011; Putman & Carbone, 2014). Direct comparisons generally report improvements in assignment accuracy and precision using large SNP panels relative to small numbers of microsatellite loci (Gärke et al., 2012; Moore et al., 2014; Morin et al., 2012). However, as information content is in part a function of the number of alleles measured, on a per‐amplicon basis, the use of microsatellite loci with their multi‐allelic nature should in some contexts provide greater population resolution relative to biallelic SNPs. The main limitation associated with the use of microsatellite loci has been the laboratory‐intensive use of electrophoretic methods and the inference of genotypes from DNA fragment mobility data resulting in error rates of 1% commonly reported (Ellis et al., 2011; Hess et al., 2012; Kelly, Mateus‐Pinilla, Douglas, Shelton, & Novakofski, 2011). However, advances in DNA sequencing, and the development of bioinformatics pipelines such as MEGASAT (Zhan et al., 2017) to score microsatellite loci from sequence data, allow large numbers of loci and individuals to be simultaneously genotyped, a task that was previously impracticable. Moreover, initial estimates of genotyping error of sequenced microsatellites suggest an order of magnitude reduction in comparison to electrophoretic approaches (Zhan et al., 2017). This in turn offers the potential for dramatic improvements in both population resolution and assignment accuracy that have been heretofore unattainable.

Atlantic salmon, *Salmo salar*, is an anadromous salmonid characterized by large‐scale ocean migrations (e.g., Dadswell, Spares, Reader, & Stokesbury, 2010), and natal homing (Feder, Egan, & Nosil, 2012; Hendry, Castric, Kinnison, & Quinn, 2004). Populations are highly genetically structured, (Bourret et al., 2013; King, Kalinowski, Schill, Spidle, & Lubinski, 2001; McConnell, O'Reilly, Hamilton, Wright, & Bentzen, 1995), and large differences in the scale and degree of structuring are commonly observed both within and among geographic regions (Dionne, Caron, Dodson, & Bernatchez, 2008). The conservation and management of Atlantic salmon populations requires the accurate identification of populations to delineate management units (Bradbury et al., 2014, 2015; Bradbury, Hamilton, Chaput et al., 2016; Moore et al., 2014). Currently, microsatellite and SNP‐based examinations have identified 13–20 regional groups in the northwest Atlantic for assignment and mixed‐stock application (Bradbury et al., 2015; Gauthier‐Ouellet, Dionne, Caron, King, & Bernatchez, 2009; Moore et al., 2014). Of these groups, the northern range limit in Labrador is perhaps least well defined with only three regional groups across 700 km of coastline, and most of the region comprised of a single reporting group. Despite the fact that a variety of genetic and genomic resources exist for Atlantic salmon (Lien et al., 2016; Moore et al., 2014), for many applications, a lack of genetic spatial resolution continues to hamper conservation and management efforts (COSEWIC 2011; DFO 2013).

**Figure 1:**
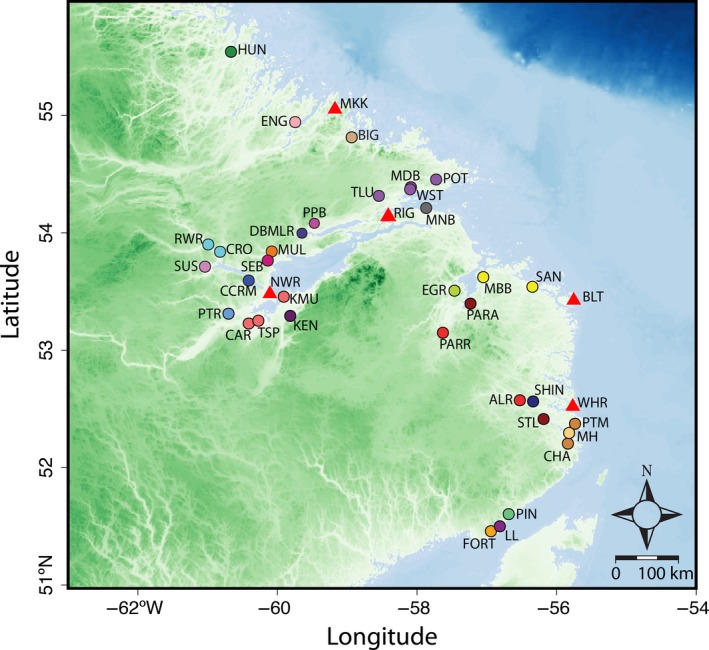
Map of Atlantic salmon baseline with reporting groups shown (colors indicate discrete reporting groups, see Figure 3) and fishery sample (red triangles) locations in Labrador, Canada. See Table 1 for sample characteristics

Our overarching goal was to evaluate the potential of sequencing large microsatellite‐based amplicon panels for fisheries and wildlife management and conservation. Our specific objective was to develop a genome‐wide microsatellite panel suited to genotyping‐by‐sequencing (GBS) which would maximize the number of alleles that could be surveyed per amplicon for individual assignment and mixture analysis in Atlantic salmon. To evaluate the resolution afforded by this methodology, we chose to focus on populations from Labrador, Canada (Figure 1), a region where genetic differentiation among populations is weak. The utility of the panel for individual assignment and mixture analysis was assessed using samples from a coastal Food, Social and Ceremonial fishery. We build upon previously developed Atlantic salmon genetic assignment studies using microsatellites (Bradbury et al., 2015; Gauthier‐Ouellet et al., 2009; Moore et al., 2014) and SNPs (Bourret et al., 2013; Moore et al., 2014) and demonstrate dramatic increases in spatial resolution of populations using our microsatellite GBS approach. We show that next‐generation sequencing of genome‐wide microsatellite loci allows large numbers of alleles to be genotyped and scored quickly and cheaply, presenting new opportunities for individual assignment and genetic mixed‐stock analysis in Atlantic salmon and other exploited species.

## 2 METHODS

### 2.1 Microsatellite discovery and testing

Microsatellite loci were identified using the ICSASG v2 genome for *Salmo salar* (Lien et al., 2016), and the program MSATCOMMANDER v. 1.0 (Faircloth, 2008). Selected loci were within size classes of 70–90, 91–110, and 111–130 bp, and had an optimum primer annealing temperature of 60°C. We targeted both trinucleotide‐motif loci with 10–13 microsatellite repeats, and dinucleotide loci with 15 repeats, and selected only one locus per genomic scaffold. Based on these selection criteria, a pool of 706 loci remained for further testing. Locus amplification followed the protocol of Zhan et al. (2017). Briefly, two sets of polymerase chain reactions (PCRs) were used, a multiplex PCR and an index PCR. Each multiplex PCR was initially performed using Qiagen's Type‐it Microsatellite PCR kit. Each oligonucleotide in the multiplex reaction was 5′‐tailed, forward oligos had one tail, and reverse oligos had a different tail. The tails included part of the Illumina sequencing primer sequence and served as oligo‐binding sites in the subsequent index PCR. PCR multiplex conditions were not optimized per locus or multiplex and included 1.75 μl Qiagen Multiplex Master Mix, 2.5–10 ng DNA, 0.35 μl Oligo Mix (1.0 μmol/L each oligo), for a total volume of 3.5 μl. Thermal reaction conditions included 95°C 15 min, 25x (94°C 30 s, 57°C 3 m, 72°C 30 s) 68°C 30 s. Multiplex‐PCR products were pooled (per sample) in equal volume amounts, then diluted 10–20 fold in water, and then used as template for the index PCR. It is worth noting, the multiplex‐PCR amplicons were not imaged or cleaned, merely pooled. The index PCR used oligonucleotides which included the Illumina annealing adapter sequence (to allow the PCR product to bind to the flow cell), a unique six‐base index (=barcode), and the sequencing primer sequence. Each sample was uniquely identified within a pooled library based on the paired six‐base indices added during this indexing PCR.

Of the microsatellites identified, we examined 282 trinucleotide loci, 96 dinucleotide loci, six previously published microsatellite loci, and one gene‐associated microsatellite locus (Vgll), using multiplexes of 24 and 48 loci. Sequencing was conducted on an Illumina MiSeq using v3 chemistry, with a target depth of 900 reads per individual per locus. Following sequencing, MEGASAT was used to demultiplex loci per individual. Each locus was examined to verify and update the input primer file, ensuring that only correct sequences were retained and only nontarget sequences were discarded. Loci were screened and were rejected if they met any of the following criteria: (i) the presence of null alleles, (ii) low variability in test samples (i.e., <4 alleles), (iii) poor amplification (loci which were under represented (<200 depth/sample) or over represented (>10,000 depth/sample), (iv) >2 alleles per individual indicative of region of genomic duplication (e.g., Lien et al., 2016) with insufficient variation in the flanking region to identify the nontarget locus, (v) difficulty scoring using MEGASAT due to excessive stutter or weakly amplifying alleles. By beginning with hundreds of loci, we were able to discard every locus that was even minimally poor by any of our criteria. A final panel of 101 loci (two previously published, 15 dinucleotide, 84 trinucleotide) was accepted for further genotyping (see Table S1). These loci were heuristically divided into four multiplex PCRs (31, 30, 20, 20 loci per multiplex). Estimates of genotyping error were made using comparisons of redundant and control samples (*n* = 56, see below).

### 2.2 Labrador baseline samples

Baseline samples were collected from 2009 to 2015 and encompassed 1,558 individuals from 35 rivers (See Table 1) across Labrador (Figure 1). Sample sizes averaged 44 individuals per river, and ranged from 13 to 50 individuals (Table 1). Fin clips were collected either via electrofishing or angling in rivers or brooks. Multiple cohorts and locations were selected in each river to avoid sampling‐related individuals when possible. DNA was extracted from both fishery and baseline samples using the Qiagen DNeasy 96 Blood and Tissue extraction kit (Qiagen) following the guidelines of the manufacturer. DNA was quantified using QuantIT PicoGreen (Life Technologies) and diluted to a final concentration of 10 ng/μl in 10 mmol/L Tris (Buffer EB, Qiagen), and microsatellite loci were amplified and genotyped as per above.

**Table 1:** Rivers from Labrador, Canada, and their associated code, reporting group, sample sizes, and type of sample (river or fishery)

Location	Code	Sample	Year	Reporting group	Type
Hunt River	HUN	49	2014	HUN	River
English River	ENG	60	2010	ENG	River
Big River	BIG	50	2009	BIG	River
Pottle's Bay	POT	13	2016	POTMDBTLUWST	River
West Brook	WST	31	2016	POTMDBTLUWST	River
Middle Brook	MDB	50	2016	POTMDBTLUWST	River
Tom Luscombe	TLU	50	2016	POTMDBTLUWST	River
Partridge Point	PPB	50	2016	PPB	River
Double Mer	DBMLR	50	2016	DBMLR	River
Mulligan River	MUL	50	2014	MUL	River
Sebaskatchu River	SEB	30	2014	SEB	River
Crooked River	CRO	51	2014	CRORWR	River
Red Wine River	RWR	50	2014	CRORWR	River
Susan River	SUS	50	2014	SUS	River
Cape Caribou	CCRM	42	2014	CCRM	River
Caroline River	CAR	25	2014	CARTSPKENKMU	River
Traverspine River	TSP	50	2014	CARTSPKENKMU	River
Kenamich River	KEN	30	2016	CARTSPKENKMU	River
Kenamu River	KMU	18	2014	CARTSPKENKMU	River
Peters River	PTR	52	2014	PTR	River
Main Brook	MNB	42	2014	MNB	River
Eagle River	EGR	50	2011	EGR	River
Paradise River	PARR	40	2011	ALRPARR	River
Paradise Brook	PARA	42	2011	PARA	River
Muddy Bay Brook	MBB	50	2011	MBBSAN	River
Sand Hill	SAN	50	2010	MBBSAN	River
Alexis River	ALR	50	2009	ALRPARR	River
Shinny's River	SHIN	50	2011	SHIN	River
St. Lewis River	STL	50	2011	STL	River
Port Marnum	PTM	33	2011	CHAPTM	River
Mary's Harbour	MH	50	2011	MH	River
Charles River	CHA	50	2011	CHAPTM	River
Pinware River	PIN	50	2010	PIN	River
L'anse au Loop	LL	50	2011	LL	River
Forteau River	FORT	50	2011	FORT	River
Makkovik River	MKK	44	2011–2016	MKK	Fishery
Upper Lake Melville/NWR	NWR	441	2011–2016	NWR	Fishery
Rigolet	RIG	63	2015	RIG	Fishery
Williams Harbour	WLH	27	2011–2016	WLH	Fishery
Black Tickle	BLT	47	2011–2016	BLT	Fishery

### 2.3 Baseline reporting groups and assignment accuracy

Reporting groups (i.e., populations for assignment purposes, Kalinowski, Manlove, & Taper, 2007) were identified using an iterative process. Reporting groups consisted of single‐population samples or lumped populations (small and/or similar samples) designed to maximize overall and reporting group assignment accuracy. Lumping of samples to form reporting groups where necessary was based primarily on a genetic distance‐based neighbor‐joining tree (i.e., Cavalli‐Sforza and Edwards distance), but also considered the distribution of mis‐assignments and geographic proximity. Small samples (three locations, *n* < 30) were combined with other locations and represent regional reporting groups. We conducted both individual self‐assignment and mixture simulations based on our defined reporting groups following Hasselman et al. (2015) using the R package RUBIAS (Anderson, 2017). RUBIAS is a Bayesian hierarchical genetic stock identification approach which accounts for population structure and differences in the number of populations grouped into baseline reporting units. We first conducted baseline individual assignment simulations using a leave‐one‐out procedure (Anderson, Waples, & Kalinowski, 2008) to estimate the accuracy and efficiency of the reporting units. Next, 100% simulations, in which mixtures are simulated where 100% of the individuals from a single reporting unit were run with 50 simulations of 100 fish per simulated mixture, and the assignment accuracy per reporting unit was determined. Second, we conducted more realistic fisheries mixtures, first using equal proportions of all reporting units (*n* = 500 individuals from each of 26 reporting units) and 20 simulations, and then comparing a range of actual to simulated proportions across 100 replicates of simulated mixtures each consisting of 500 individuals. The realistic proportions for each baseline were created by simulating with all parameters equal to 1.5 from a Dirichlet distribution. All mixture simulations also used a leave‐one‐out approach and employed a flat Dirichlet distribution. Use of Dirichlet simulation is superior to either self‐assignment or 100% simulation methods as it allows observation and determination of biases and precision over a realistic range of stock proportions (Moran and Anderson in prep). However, we include and report the results of both self‐assignment and 100% simulations here as they are commonly used in fisheries contexts.

Accuracy and efficiency for individual assignment analysis were evaluated using self‐assignment testing. Here, accuracy is defined as the proportion of the mixture or individuals correctly assigned to a reporting group (i.e., # correctly assigned/total # assigned to group), and efficiency relates to the number of individuals known to belong to a reporting group which were recovered from the mixture (i.e., # individuals correctly assigned to reporting group/# known a priori to belong to reporting group; Vähä & Primmer, 2006). For our analyses, the Bayesian posterior probability of assignment threshold was set to 0.70 to minimize the potential for type I error (following Vähä et al., 2011). For both the 100% simulation and the tests of realistic fishery mixture proportions, their efficacies were determined by comparing the known simulated proportions for each reporting group, to the proportions returned by RUBIAS. We also evaluated the accuracy provided by subsets of these loci. Here, 100% simulations were also conducted using only the loci in the PCR multiplex that displayed the highest accuracy alone, and accuracy was compared to the additional of the loci in the remaining multiplexes.

### 2.4 Fishery analysis

Fishery tissue sampling was conducted in conjunction with NunatuKavut Community Council fisheries guardians and conservations officers of the Nunatsiavut Government between 2011 and 2014. The Labrador FSC (Food, Social, and Ceremonial) fishery is composed of four subsistence fisheries harvesting Atlantic salmon along the Labrador coast: (i) Nunatsiavut Government members fishing in northern Labrador coastal communities and Lake Melville; (ii) the Innu Nation members fishing in northern Labrador and Lake Melville; (iii) Labrador residents fishing in Lake Melville and coastal communities in southern Labrador, and (iv) the NunatuKavut Community Council (NCC) members also fishing in southern Labrador (Figure 1). Mixed‐stock analysis was performed on 622 Atlantic salmon divided into five fishery samples representing general regions (Table 1), and not necessarily indicative of exact location of harvest which is not always available.

Composition of these FSC Fishery samples was estimated using a Bayesian approach with parametric bootstrapping in *RUBIAS*. For each fishery, we used 20,000 MCMC iterations in RUBIAS with the first 1,000 iterations discarded as burn‐in, along with 100 parametric bootstrap correction iterations, to obtain the individual assignments and overall proportions of each mixed‐stock fishery. Hasselman et al. (2015) have shown that use of the parametric bootstrap correction as implemented in RUBIAS reduces biases in mixed‐stock estimates by >50%. For each fishery sample, we estimate the proportion contributed from each reporting unit, and the 95% credible interval from MCMC samples of the mixing proportions of the posterior probability distribution. The Bayesian GSI method employed here has been shown to accommodate the relatively small sample sizes seen in some of the FSC fisheries, and differences in sample size are reflected in the width of the 95% credible intervals.

## 3 RESULTS

### 3.1 Microsatellite discovery and testing

**Figure 2:**
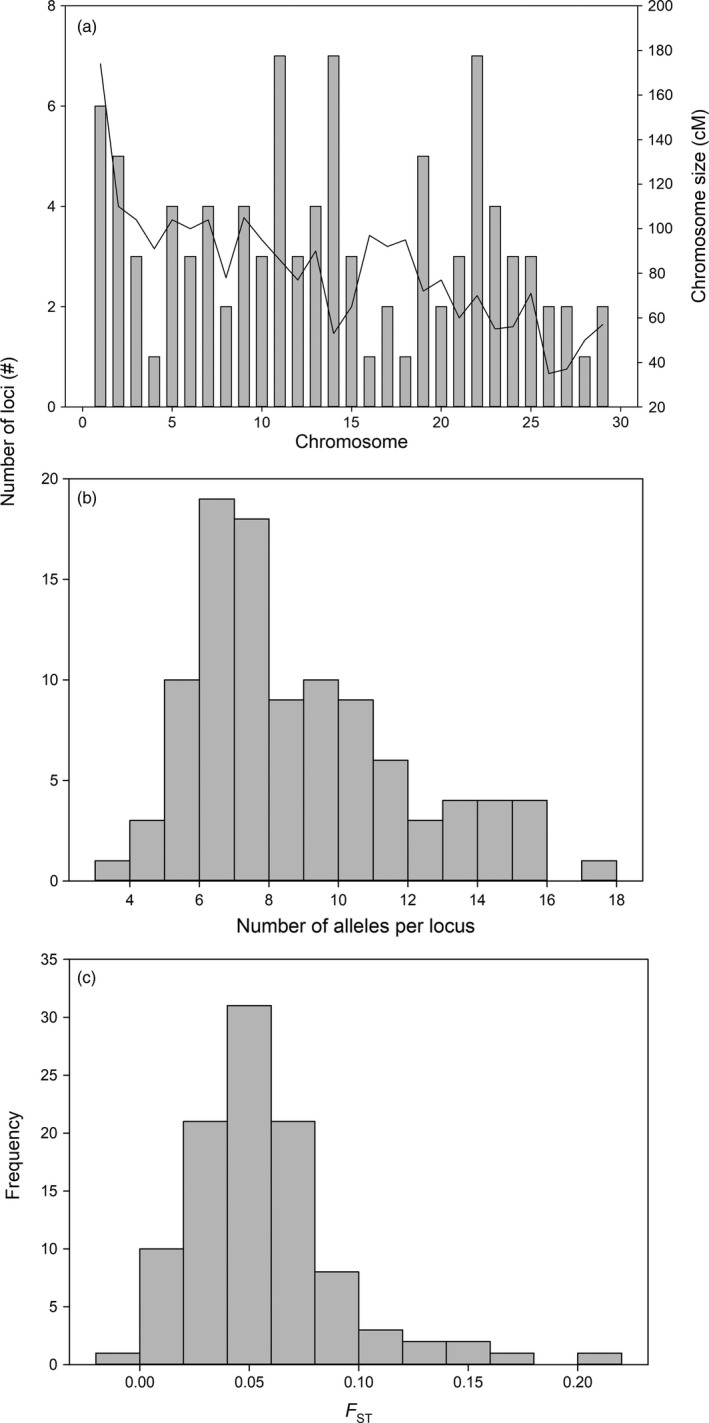
(a) Distribution of assayed microsatellite loci across the Atlantic salmon genome (gray bars) and size of each chromosome (solid line); (b) frequency distribution of number of alleles per locus using baseline samples of Atlantic Salmon from Labrador; and (c) frequency distribution of locus‐specific *F*_ST_ using baseline samples of Atlantic salmon from Labrador

The final panel of 101 microsatellites (two previously published, 15 dinucleotide, 84 trinucleotide) included loci distributed across the genome (see Table S1), with one to seven (average 3.4) loci per linkage group (Figure 2a). Numbers of final loci per linkage group were not significantly correlated with linkage group size (*p* > .05, Figure 2a). The panel was applied to samples from 35 Atlantic salmon populations (*n* = 1485 individuals after removal of individuals with >50% missing data) from coastal Labrador, Canada, and identified a total of 844 alleles with an average of 8.4 alleles per locus (Figure 2b). Observed and expected heterozygosity were 0.497 and 0.528, respectively. Tests for deviation from Hardy–Weinberg equilibrium revealed that ~1% (42 of 3,535) of population/locus comparisons were significant after Bonferroni correction for multiple tests (~8.5% of comparisons before correction) and generally significant comparisons were distributed across loci and populations (Figure S1). Pairwise *F*_ST_ values ranged from 0.151 to 0.001 with an average of 0.051, and all comparisons were significant at *p* < .001 (Figure 2c). Examinations of genotyping error using this panel revealed a total of 16 errors across the 101 loci for the 56 repeated individuals with a maximum of four errors observed at a single locus. Based on this, the genotyping error is approximately 0.14% using this microsatellite panel.

### 3.2 Baseline reporting groups and assignment accuracy

**Figure 3:**
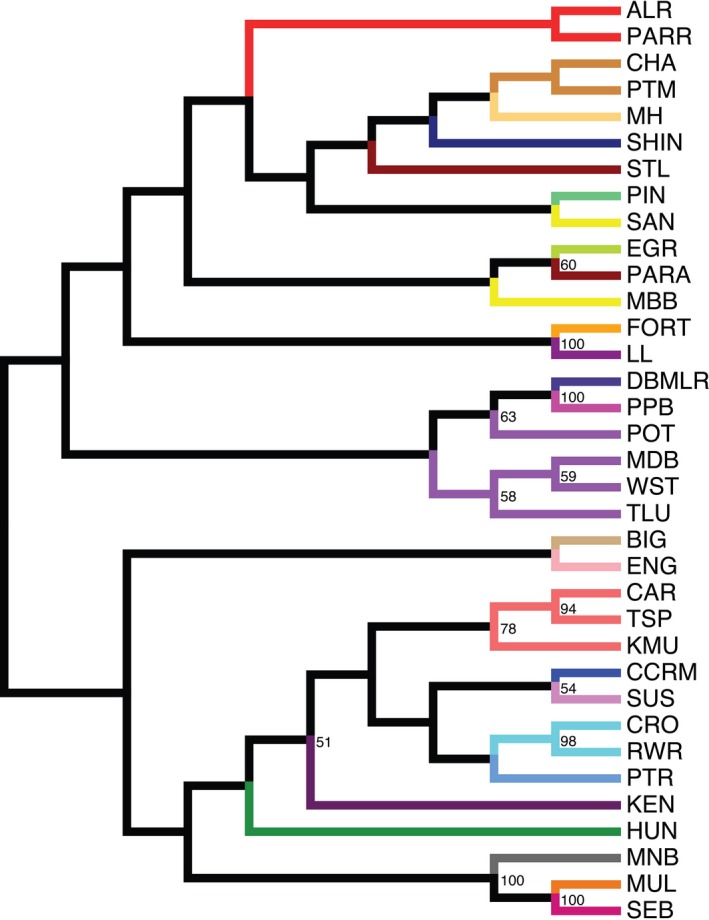
Neighbor‐joining tree of sequenced microsatellite baseline for Labrador Atlantic salmon. Colored branches correspond to reporting groups (see Figure 1). Values on the tree represent bootstrap values >50%

Initial assessments of assignment accuracy were conducted using each river sample (*n* = 35) as a single reporting group, but preliminary and previous analyses (Sylvester et al., 2017) indicated some clustering was required for accurate assignment. The genetic distance‐based neighbor‐joining tree revealed clear regional structuring with a southern cluster, a Lake Melville/north cluster, and group representing coastal locations near the mouth of Lake Melville (Figure 3). Clustering of locations largely based on this tree and mis‐assignments was required and reduced the number of reporting groups (RG) from 35 to 26 (Figure 3). As such, some of the reporting groups were combined as follows: (i) the Crooked/Red Wine RG; (ii) the Caroline/Traverspine/Kenamu Rivers RG; (iii) the Pottles Brook/Middle Brook/Tom Luscombe River/West Brook RG; (iv) the Alexis/Paradise Rivers RG; (v) the Muddy Bay/Sand Hill Rivers RG; (vi) the Charles/Port Marnum Rivers RG. Clustering generally lumped adjacent rivers except for Alexis and Paradise Rivers, which although not adjacent, have watersheds that are in close proximity to one another. The exception to this was Muddy Bay and Sandhill Rivers. Despite being disparate on the tree, common mis‐assignment between these locations supported combining into a reporting group.

**Figure 4:**
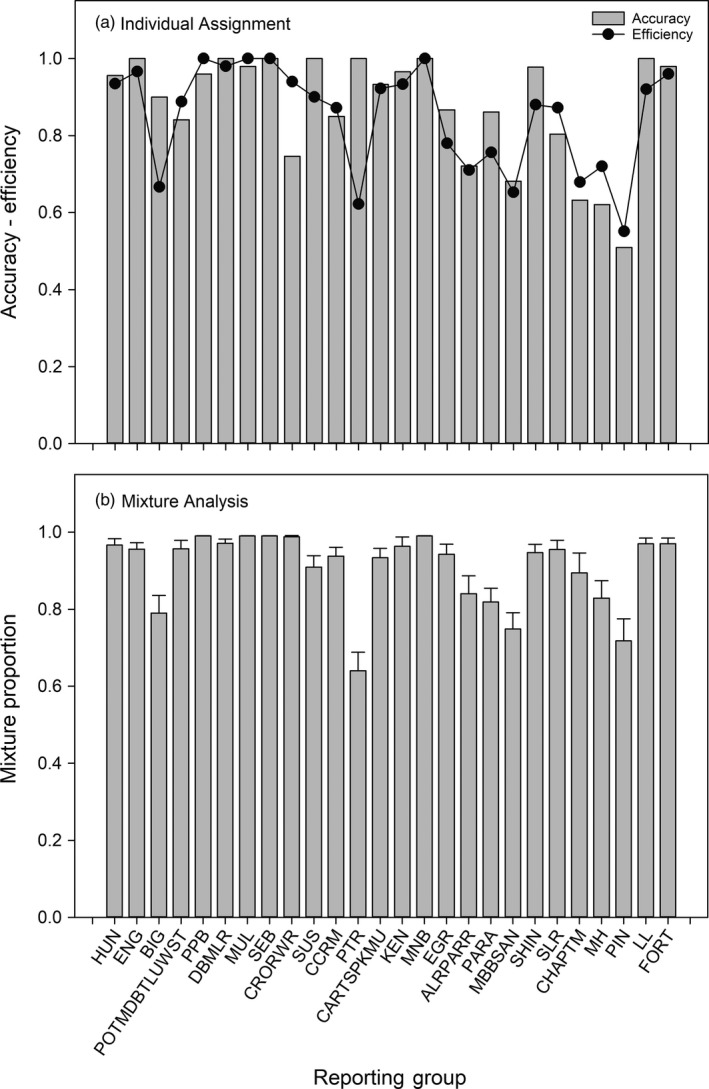
(a) Individual assignment accuracy and efficiency of Atlantic salmon in Labrador, Canada, to 26 regional groups based on a panel of genome‐wide microsatellites. (b) 100% mixture simulations for Atlantic salmon in Labrador, Canada, to 26 regional groups based on a panel of genome‐wide microsatellites. See Methods for details regarding the calculation of accuracy and efficiency and Figure 1 and Table 1 for definition of reporting groups

**Figure 5:**
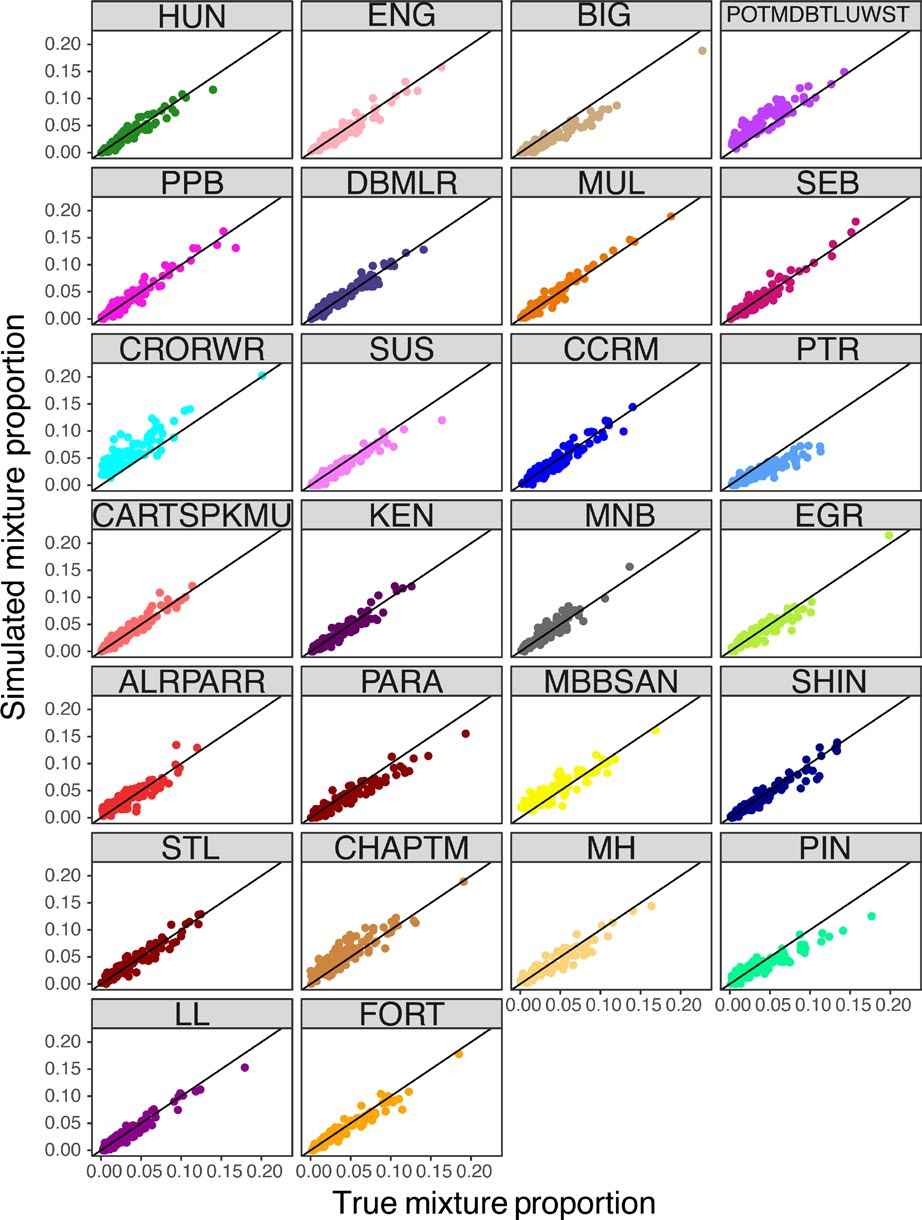
Comparison of true simulated mixture proportions and estimated mixture contributions for each of the 26 reporting groups. See Methods for details and Figure 1 and Table 1 for definition of reporting groups

Simulations were used to assess the accuracy of both mixture and individual assignment and revealed high accuracy to reporting group. For the 100% simulations, individual assignment accuracy was evaluated overall and by incrementally adding loci in each of the four multiplexes. Using subsets of loci (Figure S2), the accuracy was <70% when using the single best multiplex and increased with additional loci. Using the complete panel (i.e., 101 loci), this increased to an average accuracy of 90.8%, efficiency of 89.2%, and >90% of individuals were retained at a probability threshold of 0.70 (Figure S2, Figure 4a). Accuracy was lowest for southern coastal locations including Pinware River, the Charles/Port Marnum Rivers RG, and Mary's Harbour River (~59%). Mixture analysis accuracy was generally similar, with an average of 91%, and ranged from 64% to 100% (Figure 4b). The simulation of realistic mixtures demonstrated high accuracy across the 26 reporting groups (Figure 5). Evidence for slight upward biases was present in the Crooked/Red Wine Rivers RG, while slight downward biases were apparent in Big River, Peter's River, and Pinware River. These biases are likely associated with weak differentiation among samples or low sample sizes, are minimal with respect to overall proportions, and have a modest impact on overall accuracy and efficiency (Figure 5).

### 3.3 Fishery analysis

**Figure 6:**
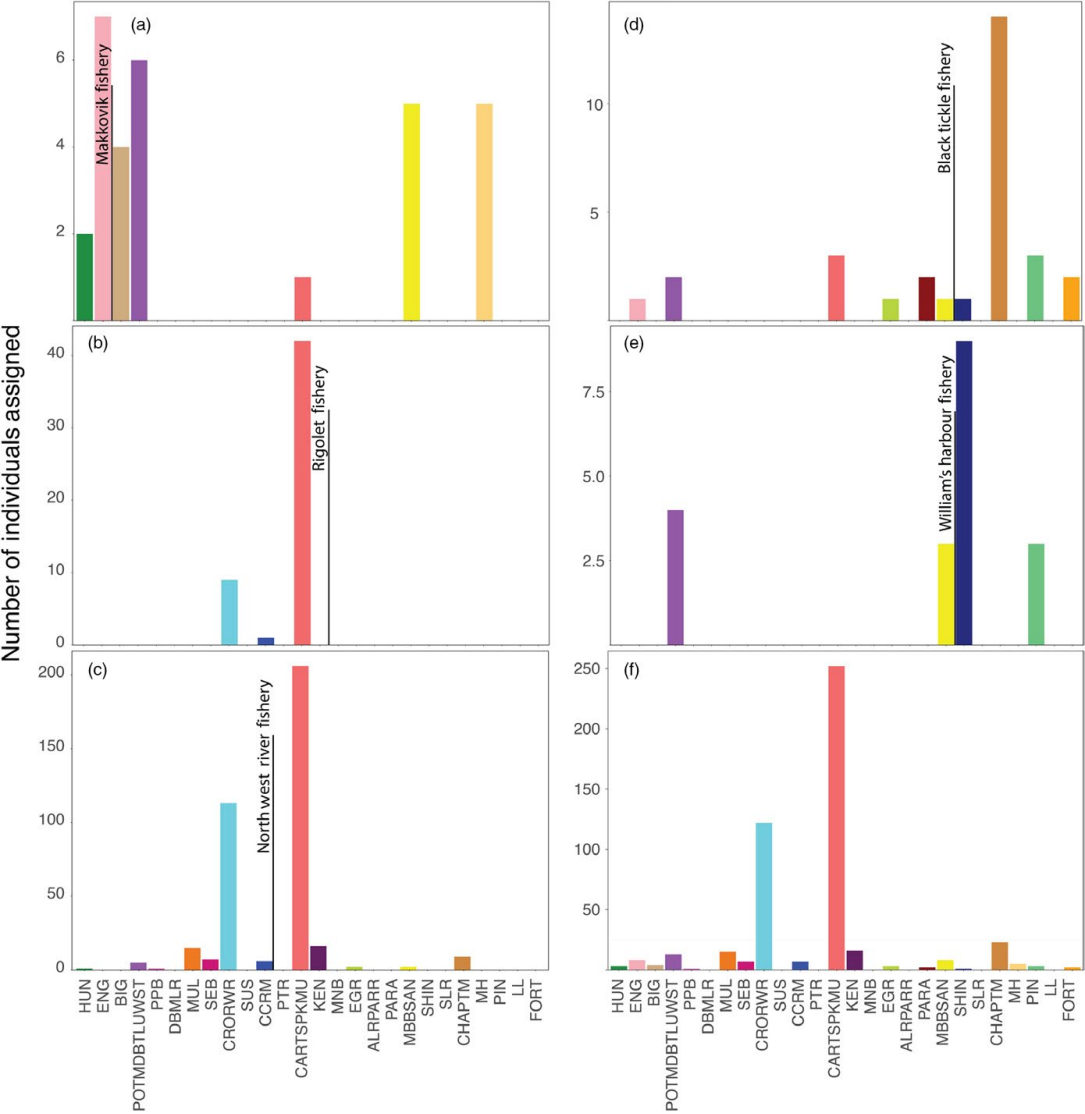
Individual assignment of Atlantic salmon from five fishery samples in Labrador (a‐e) and overall (f), to 26 regional groups based on a panel of genome‐wide microsatellites. Black lines represent relative location of the fishery sample to baseline reporting groups. See Methods for details regarding individual assignment and Figure 1 and Table 1 for definition of reporting groups

Using this baseline, we estimated reporting‐group contributions to five fishery samples collected as part of the Labrador FSC in 2011 and 2014 distributed across the region (Figure 1, Table 1). Of the 44 individuals sampled from the northernmost fishery sample, Makkovik, significant contributions were detected from coastal locations both in the north and south as well as a single individual assigning to Lake Melville reporting groups (Figure 6). For the two fishery samples from within Lake Melville (i.e., Rigolet and upper Lake Melville/NWR), catch composition was dominated by two reporting groups, Crooked/Red Wine (CRORWR) and the Caroline/Traverspine/Kenamu Rivers (CARTSPKNU), both from within the lake. Interestingly, coastal salmon were only detected in the large upper Lake Melville sample (*n* = 446), though admittedly in low numbers. Both fishery samples from southern coastal Labrador, Black Tickle and Williams Harbor, again displayed a mix of coastal locations with contributions spanning much of the coastal area surveyed. For fishery samples with adjacent reporting groups, catches were generally dominated by these groups suggesting a tendency for the fishery to target local populations. Overall, most individuals were harvested in close proximity (<300 km) to their natal river or reporting group (Figure S3). The one exception was the sample from Rigolet, which contained individuals assigned to the western end of Lake Melville (Figure S3). As Rigolet marks the entrance to this inland fjord, the fishery seems to largely target salmon migrating inland.

## 4 DISCUSSION

The resilience and stability of both species and fisheries are associated with the preservation of intraspecific variation (Hilborn et al., 2003; Schindler et al., 2010). Accordingly, fisheries targeting mixtures of populations may overexploit less‐abundant components and pose ongoing challenges for fisheries management. Genetic‐based individual assignment and mixture analysis have been used extensively to quantify stock‐specific levels of exploitation and for the management of mixed‐stock harvests (e.g., Bradbury et al., 2015; Bradbury, Hamilton, Chaput et al., 2016). Here, we demonstrate the utility of large sequenced microsatellite panels for population identification and individual assignment. Sequencing of microsatellite panels eliminates the reliance on electrophoretic methods for genotyping, thereby dramatically increasing the number of loci that can be surveyed, and greatly reducing manual labor (Zhan et al., 2017). This work extends previous applications of genetic and genomic approaches to resolve regional Atlantic salmon populations (Bourret et al., 2013; Bradbury et al., 2015; Moore et al., 2014) and to disentangle contributions to mixed‐stock harvests (Bradbury, Hamilton, Chaput et al., 2016; Bradbury, Hamilton, Chaput et al., 2016; Gauthier‐Ouellet et al., 2009) in the northwest Atlantic. We demonstrate unprecedented resolution of geographically fine‐scale reporting groups in Atlantic salmon based on only four multiplex PCRs per fish. This work suggests that the development and application of large sequenced microsatellite panels presents significant potential for stock resolution in Atlantic salmon and more broadly in other exploited anadromous and marine species.

Although microsatellites have been used for fishery assignment and mixture analysis for decades (Bradbury et al., 2015; Narum et al., 2008; Shaklee, Beacham, Seeb, & White, 1999), the use of single nucleotide polymorphisms (SNPs) has become more common (Larson et al., 2014; McKinney, Seeb, & Seeb, 2017). Recent simulation studies suggest enhanced assignment accuracy with the use of 100s to 1000's of SNPs relative to 10–20 microsatellite loci that were previously the norm for GSI (Candy et al., 2015; Moore et al., 2014; Puckett & Eggert, 2016). However, these comparisons are rarely expressed on a per‐amplicon basis, where the use of multi‐allelic microsatellite loci should maximize information content and assignment power. Using a microsatellite panel of 101 loci, we resolved 844 alleles in Labrador salmon populations, approximately seven times the number of informative alleles expected from a similarly sized SNP panel. The cost of sequencing this panel was approximately $0.07 USD per genotype or ~$7 an individual, similar to what has been reported previously (Zhan et al., 2017), and compares favorably with the cost of SNP genotyping. In comparison, a nonsequenced microsatellite dataset representing a subset of these populations contained 15 loci and resolved 389 alleles (~46% of that reported here). Despite our attempt to include more of these previously published, highly polymorphic loci in our sequenced panel, all but one were eliminated due to size and read length limitations. Future advances in read length would allow more of these loci to be added and would likely enhance the existing panel. It is important to also acknowledge that ascertainment processes or biases independent of number of alleles could also influence population resolution, and power may not be a direct function of number of alleles in all cases.

Previous attempts to identify genetically resolved reporting groups for Atlantic salmon in eastern North America have identified 12–15 regional groups for which accurate individual assignment is possible using either microsatellites (Bradbury et al., 2015; Moore et al., 2014) or SNPs (Moore et al., 2014). More recent attempts to develop a range‐wide SNP baseline have refined the number of reporting groups in North America to 20 (Jeffery et al. in review), yet only three reporting groups could be accurately resolved in Labrador. The use of our large sequenced microsatellite panel has dramatically increased the spatial resolution of reporting groups in this poorly differentiated portion of the species’ range. For comparison, the geographic scale of reporting groups (i.e., based on 15 microsatellite loci) in Bradbury et al. (2015) for North America suggested that each group encompassed an average 700 km of coastline, providing a stark contrast to the present baseline which contains 26 reporting groups within ~500 km of coastline. With this increased resolution, assignment accuracy has remained high, averaging 88%–91% for individual assignment or mixture analysis, respectively, on par with previous estimates of regional assignment accuracy in salmon populations in eastern North America (Bradbury et al., 2015; Gauthier‐Ouellet et al., 2009; Moore et al., 2014). Notwithstanding the high overall accuracy reported here, slight systematic biases either upward or downward were detected in assignment among a few reporting groups as has been noted elsewhere (Hasselman et al., 2015), but these biases seem to have minimal impact on the levels of overall accuracy.

Our limited analysis of the fishery samples revealed a tendency for harvest composition to be dominated by adjacent populations and extends previous conclusions of localized exploitation in this region (Bradbury et al., 2015). This was particularly true of catches from within Lake Melville, which were dominated by two reporting groups from within the lake. Despite this trend, there were assignments among coastal regions, suggesting that some movement occurs. The scale of movement is consistent with regional groups identified using SNP‐based assessments of stock structure in the region and the clear isolation of populations within Lake Melville from coastal groups (Sylvester et al. in preparation). These results support previous conclusions using small microsatellite‐based panels and tagging results that this fishery largely targets Atlantic salmon of Labrador origin (Bradbury et al., 2015; Pippy, 1982). It is worth noting that our inclusion of fishery samples here was to demonstrate the utility of sequenced microsatellite panels for analysis of mixed‐stock harvests and does not represent a quantitative analysis of exploitation in this fishery. Admittedly, additional examination will be required to accurately measure stock‐associated exploitation including an analysis of temporal stability of this baseline given samples were collected over several years. Moreover, as several reporting groups used here displayed accuracies of <80% in the mixture analysis, some refinement of reporting groups may be justified depending on the analysis. Further comparisons between sequenced microsatellite and SNP haplotype‐based panels are warranted, given recent evidence that amplicon haplotypes dramatically improve assignment power over SNP panels (McKinney et al., 2017).

## 5 CONCLUSIONS

Mixed‐stock fisheries require management techniques that depend on the knowledge of stock‐specific exploitation. Here, we show that a genome‐wide panel of sequenced microsatellite loci can provide dramatic improvements in the resolution of stock structure and revealed fine‐scale differences in composition in an Atlantic salmon fishery in Labrador. More broadly, the development and application of large sequenced microsatellite panels presents unprecedented potential for stock resolution in Atlantic salmon and more broadly in other exploited anadromous and marine species. It is likely that additional analysis of both baseline samples and fishery catches could further enhance this baseline and reveal further spatial and temporal variation in catch composition. This work directly extends a recently developed SNP baseline that provides range‐wide resolution of North American stocks but only coarse fine‐scale stock discrimination (Moore et al., 2014). Harvests targeting mixtures of stocks in Atlantic salmon and other species continue to complicate fisheries management and threaten population and fishery stability (Hilborn et al., 2003; Schindler et al., 2010). The abundance of Atlantic salmon in many regions of North America has been declining in recent years (ICES 2015), and marine mortality has been identified as the dominant challenge to sustainability. As such this work represents a significant advance in our ability to identify populations, quantify fishery‐associated exploitation at sea, and effectively manage and conserve exploited species.

## CONFLICT OF INTEREST

None declared.

## DATA ARCHIVING STATEMENT

Data for this study are available in the Dryad Digital repository at: https://doi.org/doi:10.5061/dryad.864rv
