# Supplementary material for: Genotyping‐by‐sequencing of genome‐wide microsatellite loci reveals fine‐scale harvest composition in a coastal Atlantic salmon fishery
Source: Evol Appl. 2018 Mar 11;11(6):918–30. doi: 10.1111/eva.12606 (PMC5999200; doi:10.1111/eva.12606)
Supplement: Supplementary file 1 [file EVA-11-918-s001.docx]

**Supplemental Information for**

**Genotyping-by-sequencing of genome-wide microsatellite loci reveals fine-scale harvest composition in a coastal Atlantic salmon fishery**

Table S1. Locus name, primers, and repeat motif for microsatellite loci used. Previously published loci are the first two listed. New loci are named using the following system: *Salmo salar* -Chromosome#.locus#. PCR multiplex conditions: Qiagen Multiplex Master Mix:  1.75ul, DNA (2.5-10ng): 1.4ul; Oligo Mix (1.0uM each oligo): 0.35ul, Total volume 3.5ul, 95C 15min, 25x(94C 30s, 57C 3m, 72C 30s) 68C 30.

| **Locus Name** | **Multiplex** | **left Oligo** | **Right Oligo** | **Repeat Motif** | **Chromosome** | **NCBI Accession #** |
| --- | --- | --- | --- | --- | --- | --- |
| NGS-SsaD486 | 1a | TGCAGTCCAATAATATCCCCGT | GTTATCCGAGTCATGCAGGG | AGAT | unknown | AF525208.1 |
| NGS-SSsp2210 | 1a | CACATTCACTGCAAAATAAAGCT | ACTTACTTACCTTTATTGAATCCCA | AGTT | unknown | AY081808.1 |
| Ssa-1.5 | 1a | GCGTTATGTGCTTGCATGC | GGATAAGCTGAGTACGGTGGT | ATT | 1 | gi\|925168832\|ref\|NW_012332749.1\| |
| Ssa-1.7 | 1a | AGAACACAACAGAACCAGGTAC | GGGTTGGAAGTGTGTTCGAG | GAT | 1 | gi\|925169069\|ref\|NW_012332512.1\| |
| Ssa-1.8 | 1a | AGGCCAAAGAAATCCTGCAC | CTATTTGCGTGTTTGGGTCAGT | ATC | 1 | gi\|925168775\|ref\|NW_012332806.1\| |
| Ssa-1.9 | 2b | CTGAGGAGCACAAAGGACAG | GAGAACACAGCCAGCAACAC | AGG | 1 | gi\|925169130\|ref\|NW_012332451.1\| |
| Ssa-1.10 | 1b | TGGATGACAACCTCCGTTAAAC | GATCTTCACCAGGCTTCCCG | AAG | 1 | gi\|925169083\|ref\|NW_012332498.1\| |
| Ssa-1.11 | 1b | CTCATCAACGCTATCCTCTTCC | CACGCGGACAGATGAAAGAC | ATC | 1 | gi\|925169075\|ref\|NW_012332506.1\| |
| Ssa-1.14 | 1b | TCGTATTTGTCAAGGATGTGCC | GGGCAATACAATGGGCATCT | AGT | 1 | gi\|925168868\|ref\|NW_012332713.1\| |
| Ssa-2.1 | 1a | AGACTCCACCTGCCTTGTTC | CGCATGCTCTGACAGTGAG | ATT | 2 | gi\|925168396\|ref\|NW_012333185.1\| |
| Ssa-2.2 | 1a | TGGCCATTCTCCAGAGCTAG | CACGTACTCTCCTTTGGTGG | CTT | 2 | gi\|925168422\|ref\|NW_012333159.1\| |
| Ssa-2.7 | 1a | CCCAGACTTCCCACTCTCTATG | CCGTTCAAGGTTCTGTGTCC | CTG | 2 | gi\|925168409\|ref\|NW_012333172.1\| |
| Ssa-2.12 | 1b | CAGTACAGAAGCAGTCATCGC | CATGACCGTCCGCAAACAAT | CTT | 2 | gi\|925168396\|ref\|NW_012333185.1\| |
| Ssa-2.13 | 1b | GCTCAGATCGCAACCTTGAC | CTCGGTCTGGTCGGTTTAGA | CCT | 2 | gi\|925168345\|ref\|NW_012333236.1\| |
| Ssa-3.2 | 2b | GTCACCAATACCACGTCACC | GTGACCACATCCCTTGACGA | ATC | 3 | gi\|925168028\|ref\|NW_012333553.1\| |
| Ssa-3.9 | 1b | CACCTCCAACTGCTCAATTAGG | GTTGAGAAACACGGGCCTC | AGT | 3 | gi\|925168037\|ref\|NW_012333544.1\| |
| Ssa-3.10 | 1b | GACTGCAACTAACTGAATGACG | CAGCTGAAAGGGATGATGGA | ATC | 3 | gi\|925168028\|ref\|NW_012333553.1\| |
| Ssa-4.d44 | 2a | TTGGGTCTTAATGGCACCTG | CACTCTCAGGGAACCAAAGC | AC | 4 | gi\|925167334\|ref\|NW_012334247.1\| |
| Ssa-5.2 | 1a | AACTTGCGTGATGATGTGGC | CACAGAAGAACATGGCCAGC | CTT | 5 | gi\|925167062\|ref\|NW_012334519.1\| |
| Ssa-5.6 | 2a | GTGCAGCTGTTCCTCACTTC | CGATTTCTACGCCTGTCCC | TAT | 5 | gi\|925167074\|ref\|NW_012334507.1\| |
| Ssa-5.8 | 2b | ACACAGCTCTTATTTAACCGTC | CTTAGACGAGTGAGATTCCTTC | AAC | 5 | gi\|925167158\|ref\|NW_012334423.1\| |
| Ssa-5.11 | 1b | CAACCGCCGTTAAACATCATC | GGCCTTCCAATTCACAATGATG | AAG | 5 | gi\|925167048\|ref\|NW_012334533.1\| |
| Ssa-6.2 | 1a | GGAGAAGAGGAGATGGAACTTG | GGTGTGGTATTGTCAGGTGT | TGA | 6 | gi\|925166267\|ref\|NW_012335314.1\| |
| Ssa-6.7 | 2a | GCAAATCAGCATTCAGGGC | CCCATTCAGTTCGATCAGCTG | TAA | 6 | gi\|925166258\|ref\|NW_012335323.1\| |
| Ssa-6.11 | 1b | CCGTGGAAAGCACTTAACATG | GAGGCCATGACATGCGTTC | ATT | 6 | gi\|925166307\|ref\|NW_012335274.1\| |
| Ssa-7.1 | 1a | CCACTCCCACGAATGATGTTC | GACTGCAATGTGGCCTCC | AAC | 7 | gi\|925166059\|ref\|NW_012335522.1\| |
| Ssa-7.12 | 2a | CACTCCCTGACACGTTAACAC | GTGCATGTTTGTCAGGAAGTG | ACT | 7 | gi\|925166045\|ref\|NW_012335536.1\| |
| Ssa-7.d33 | 2b | AGCATAGCATAGGAACAGACAC | CGATGAGGTCAGGATGTGCT | AC | 7 | gi\|925166053\|ref\|NW_012335528.1\| |
| Ssa-7.d47 | 2b | TGGAATTGGGTCAGCAGTTC | GCTGATCTCAACCCTGTCCT | GT | 7 | gi\|925166033\|ref\|NW_012335548.1\| |
| Ssa-8.d04 | 2b | ACTGTGTGGACTGGGAGATC | GGTACAAGACAACGCTGCTG | GT | 8 | gi\|925165795\|ref\|NW_012335786.1\| |
| Ssa-8.d07 | 2b | GGGTGTGAGGGAGGACTTAAC | GGACAGGAGTGTAGCTAGCA | AC | 8 | gi\|925165792\|ref\|NW_012335789.1\| |
| Ssa-9.3 | 1a | GCCAACCACCGTTAAACCTC | GGGAAATATTGGGAACTGCTGA | AAG | 9 | gi\|925165300\|ref\|NW_012336281.1\| |
| Ssa-9.8 | 1a | GCGTCGACTGCCATTCAAC | CCACGGAGAAAGCAAGGACA | AAG | 9 | gi\|925165505\|ref\|NW_012336076.1\| |
| Ssa-9.10 | 1b | TCCATTGTTCCCTCAGACCC | CCAGCTCTCCTTCATGTTACC | ATT | 9 | gi\|925165582\|ref\|NW_012335999.1\| |
| Ssa-9.13 | 2a | ATCCACACCTCTCTTGCCAC | GGGATGGTAACGATGGTGATC | AGG | 9 | gi\|925165161\|ref\|NW_012336420.1\| |
| Ssa-10.1 | 1a | GGTCCTCCAGTACCTCCAAC | CCGGACGAACTCACCAGATT | GTT | 10 | gi\|925165007\|ref\|NW_012336574.1\| |
| Ssa-10.2 | 2a | TGATCCTCTTCACCACCCTG | GGTGAGGGAGGAGTCTTCAG | AAT | 10 | gi\|925164993\|ref\|NW_012336588.1\| |
| Ssa-10.4 | 1a | GGTGAAATGTAGCCTGCATG | CCACACATATAGCAGTGTGT | AAT | 10 | gi\|925164986\|ref\|NW_012336595.1\| |
| Ssa-11.2 | 1a | AAAGTTTGTTTGTGGACCGC | GAAGTCCAAGAAACTGTCCG | AAG | 11 | gi\|925164888\|ref\|NW_012336693.1\| |
| Ssa-11.3 | 1a | AGCGTGTGTGTCGTTCAATAC | GTGACGCAGAGGTGAAACAT | AAC | 11 | gi\|925164793\|ref\|NW_012336788.1\| |
| Ssa-11.5 | 1a | GTGTGCCGTTCTATCGCTG | CGGACTCTGGCATTTCTTTAGG | GAT | 11 | gi\|925164446\|ref\|NW_012337135.1\| |
| Ssa-11.6 | 1a | TTAACCTGCTCTACCTCTCG | GAAGATAGGTGTGGTGATGT | TGA | 11 | gi\|925164433\|ref\|NW_012337148.1\| |
| Ssa-11.8 | 2a | AAAGGACCCAGAACGTACAG | CATTGAGGGTACTGTGTGGT | ATA | 11 | gi\|925164901\|ref\|NW_012336680.1\| |
| Ssa-11.11 | 1b | CGGCATATACCTTTAACGTTGG | CCTCTCGCATCGCTTCTTC | AAG | 11 | gi\|925164498\|ref\|NW_012337083.1\| |
| Ssa-11.12 | 1b | CGTTAGCACACATGGCAAATC | GATGCATCCCAAACAGCACC | ACT | 11 | gi\|925164426\|ref\|NW_012337155.1\| |
| Ssa-12.5 | 1a | TCTCCTTCCTCGATCAGCTC | GGTGGGAAGGCGACACATT | ATC | 12 | gi\|925164069\|ref\|NW_012337512.1\| |
| Ssa-12.12 | 1b | TTGCTGCTGGTTTGTGCTC | GCAATACCACTTCACTGTCCC | GAT | 12 | gi\|925164059\|ref\|NW_012337522.1\| |
| Ssa-12.13 | 1b | ATCAGGCTCAGAGGTGGAAC | GCTATCTCTACCTCCACTGTGT | CCT | 12 | gi\|925164020\|ref\|NW_012337561.1\| |
| Ssa-13.2 | 2b | CTACACCAAGAGTCCAGTGTC | CAACAACAGGGAGACAAATTGT | AAT | 13 | gi\|925163922\|ref\|NW_012337659.1\| |
| Ssa-13.8 | 2a | TGACGAGACAAGATTCAGGTTG | CGTTGGTGGTTGCATAGGTC | GTT | 13 | gi\|925163870\|ref\|NW_012337711.1\| |
| Ssa-13.10 | 1b | TGAAAGTTGGCTGCAATCCG | GTGTGGGCAGACAGGTTCC | AAG | 13 | gi\|925163859\|ref\|NW_012337722.1\| |
| Ssa-13.12 | 1b | AGTTTGGCGTAGTCTGGGAC | CCCAGAGGAGGATGATGGGA | AGG | 13 | gi\|925163769\|ref\|NW_012337812.1\| |
| Ssa-14.2 | 1a | GGGCATGATCTCGACACC | CTTAGCCAGCTTACTCATTCCT | ATC | 14 | gi\|925163728\|ref\|NW_012337853.1\| |
| Ssa-14.3 | 2a | TCAACCTAAACCCTCTGCCC | GTTGCTGTGGAATGTGATGATT | AAT | 14 | gi\|925163708\|ref\|NW_012337873.1\| |
| Ssa-14.5 | 2a | CCAGGAGGCCTTCACATG | CTATACAGCATTGCCAGGAGG | AAT | 14 | gi\|925163651\|ref\|NW_012337930.1\| |
| Ssa-14.6 | 1a | AGTCAAGAAAGTCACTGCCC | CCCTTTCTGTTTGCCATTCC | ATT | 14 | gi\|925163718\|ref\|NW_012337863.1\| |
| Ssa-14.8 | 2a | AAACATTGATTTGGCTCTGTC | GAGAACGGGATGGTGCAATA | TAT | 14 | gi\|925163650\|ref\|NW_012337931.1\| |
| Ssa-14.9 | 1b | CCATAATGGCACTGCTTCTTC | CGGAGTGTAATGAAGCAACAC | AAT | 14 | gi\|925163731\|ref\|NW_012337850.1\| |
| Ssa-14.10 | 1b | GGGAACGTGTGGAAGATTCAC | GGCATCACCCTCCATACCTT | ATC | 14 | gi\|925163717\|ref\|NW_012337864.1\| |
| Ssa-15.1 | 1a | TTTCTTTGTGTGTTGTGCCC | CCCAGAGGAACCACAGCTG | CCT | 15 | gi\|925163276\|ref\|NW_012338305.1\| |
| Ssa-15.3 | 1a | GCTAACGAATGACAGCTTGC | CTGCTGCCAGTCTTACTAATG | TTG | 15 | gi\|925163318\|ref\|NW_012338263.1\| |
| Ssa-15.7 | 1a | GATGTGATGGCAGTGCTATG | GGAGATTGACCTTGTTGCTG | TGA | 15 | gi\|925163309\|ref\|NW_012338272.1\| |
| Ssa-16.5 | 1b | CCGCTGGATTCCTCATTATGTC | GGTCTCTCTTCCTGTCAGTCC | TTA | 16 | gi\|925163075\|ref\|NW_012338506.1\| |
| Ssa-17.1 | 2a | CATCTTCCGGTTCGCTCAAC | CTGGTTGCACAGGTCATGAC | ATT | 17 | gi\|925162405\|ref\|NW_012339176.1\| |
| Ssa-17.2 | 2b | ACCCATAGAATTACTGCACTGG | GTTGACATTATGCCAGTACGAC | ATT | 17 | gi\|925162379\|ref\|NW_012339202.1\| |
| Ssa-18.7 | 2a | TGCAGGTTGTGGTCATGTTG | GGCCGAATGGACAGAATGTG | TTA | 18 | gi\|925161808\|ref\|NW_012339773.1\| |
| Ssa-19.1 | 1a | TGTGCAAACGCCATGATACC | CCGGATGGAGCTGTCATGG | GTA | 19 | gi\|925161618\|ref\|NW_012339963.1\| |
| Ssa-19.2 | 1a | GTGACCCAAAGTGCTGCTG | GAGGTGCTGGTGTCTGGAG | GCT | 19 | gi\|925161617\|ref\|NW_012339964.1\| |
| Ssa-19.3 | 1a | ACGTCCTGACAGTTATCCTTG | GAGCACAGCCATGACAAGAC | TTC | 19 | gi\|925161703\|ref\|NW_012339878.1\| |
| Ssa-19.7 | 1b | CTCCTTCACACAACCACC | CACAAGGTAGGTCTGCACTT | AGC | 19 | gi\|925161695\|ref\|NW_012339886.1\| |
| Ssa-19.9 | 1b | TCTGGTGCTGACGATGAGAG | GGGCCAATGACCTCTGATTTC | GAT | 19 | gi\|925161703\|ref\|NW_012339878.1\| |
| Ssa-20.2 | 1a | TCTTCCCTCTTCTGCAGCAG | CAGTGTGGTGTCCAGAGCT | GTG | 20 | gi\|925161392\|ref\|NW_012340189.1\| |
| Ssa-20.d56 | 2a | GAGGTCAAGGTTTCCACTGG | CCCAGAACAGAGAGCAGCTA | AG | 20 | gi\|925161350\|ref\|NW_012340231.1\| |
| Ssa-21.2 | 2a | CTGTCCAAATTGCAGGCTTG | GACAGGAGTAGGCAAATTAGGC | TAT | 21 | gi\|925161196\|ref\|NW_012340385.1\| |
| Ssa-21.3 | 1b | TTGAACCTGAACTGGAATCCC | CTGTTTCAGACTGGCCGGT | ATC | 21 | gi\|925161206\|ref\|NW_012340375.1\| |
| Ssa-21.5 | 1b | CACTCCCTAACTCCATGGTC | CACAGTGACGACATCCATGA | ATG | 21 | gi\|925161233\|ref\|NW_012340348.1\| |
| Ssa-21.10 | 1b | ACTGCTTAGCTAGATTTGGCC | GCATGTTCACCTGTCTGTAGA | ATT | 21 | gi\|925161196\|ref\|NW_012340385.1\| |
| Ssa-22.2 | 2a | AGTGGTTGCTTTGGTTCTCC | CGTCTTGGTCCGCTTTATCC | AAT | 22 | gi\|925161105\|ref\|NW_012340476.1\| |
| Ssa-22.5 | 2a | GTGACGTCTGGAATTGTGAC | CTACCGGTGTTGATTGGATC | AAT | 22 | gi\|925161127\|ref\|NW_012340454.1\| |
| Ssa-22.9 | 2a | CAAATGCCACACGACCTGAC | CTATATGCAGAGCGGTTGACC | ATT | 22 | gi\|925161127\|ref\|NW_012340454.1\| |
| Ssa-22.d31 | 2a | AGTTTAGTAGGGCCTGCGTG | CAGGCTGTGACAGAAGAATGT | GT | 22 | gi\|925161077\|ref\|NW_012340504.1\| |
| Ssa-22.d40 | 2b | GCACAGAGGTAAGAGTTCAGC | CCACCCACAGCAGCAGAG | AC | 22 | gi\|925161053\|ref\|NW_012340528.1\| |
| Ssa-22.d41 | 2b | CTCTGTGGTCTGGGTCCTC | GATGTGCATGGGTACGAGGT | CT | 22 | gi\|925161051\|ref\|NW_012340530.1\| |
| Ssa-22.d44 | 2b | GTACCTTTGAACATGCACACG | GCAACGTTATCATGTGGAGATG | AC | 22 | gi\|925161034\|ref\|NW_012340547.1\| |
| Ssa-23.2 | 1a | GGTGGTTGTTTCTAGTGAGGG | CCATGGTGCTTTAGAGGTGC | CTT | 23 | gi\|925161018\|ref\|NW_012340563.1\| |
| Ssa-23.3 | 1b | GGAGAAGTGATTATGGTTGTGC | CCATGTAGAACCCGTTGTCC | AAC | 23 | gi\|925160997\|ref\|NW_012340584.1\| |
| Ssa-23.9 | 1b | ACGGATACAGAGAGACGCAC | GACTTTGTCCTCCTCGCTGT | ATC | 23 | gi\|925161018\|ref\|NW_012340563.1\| |
| Ssa-23.10 | 1b | TGATTGTGAACGGCTTTGGG | GACAAAGGGTGCTTGCTTGT | GAT | 23 | gi\|925161012\|ref\|NW_012340569.1\| |
| Ssa-24.9 | 1b | CACTCCATCTATCATCTGTGCC | GGCCTCTTCTGCTCCTCATC | CTT | 24 | gi\|925160923\|ref\|NW_012340658.1\| |
| Ssa-24.d09 | 2b | ACCGTAAGCAGCATCACTTTAG | CAGTACCAGACAGCCCAAAC | AC | 24 | gi\|925160918\|ref\|NW_012340663.1\| |
| Ssa-24.d24 | 2b | CTGCCAACACACACTGCC | CCGACATACAGGAAGAGTCAAA | CT | 24 | gi\|925160864\|ref\|NW_012340717.1\| |
| Ssa-25.2 | 1a | TGCAGGAAGACTCTGAAAGG | CTGATGTACAACACCCACCT | GAT | 25 | gi\|925160771\|ref\|NW_012340810.1\| |
| Ssa-25.3 | 1b | TTCCCACTGGCCAAGAACTG | GTCATCAACACAAGGGAATGTC | AAC | 25 | gi\|925160672\|ref\|NW_012340909.1\| |
| Ssa-25.11 | 2a | GGGTCCATGAGAAAGGCAAC | GTTGTCAGGTGTGGATCCCA | ATT | 25 | gi\|925160727\|ref\|NW_012340854.1\| |
| Ssa-26.1 | 2a | TCACGCATAACCTTAGACAACC | GCTGTAACAGGGTTGGCATT | ATT | 26 | gi\|925160353\|ref\|NW_012341228.1\| |
| Ssa-26.d06 | 2b | CATAATCACCTTGCATGACACC | GTATTTAGCGGTGCAGCAGG | AC | 26 | gi\|925160645\|ref\|NW_012340936.1\| |
| Ssa-27.7 | 1b | TCATCAGTGTGGAGGGAATC | CCAGGCCAGAGGAAGATAGA | TTG | 27 | gi\|925160107\|ref\|NW_012341474.1\| |
| Ssa-27.d46 | 2b | TGGCTGGTGGTTATAGGAGC | CATCACTGTCTTGGCATGGT | AC | 27 | gi\|925160006\|ref\|NW_012341575.1\| |
| Ssa-28.d01 | 2b | ATTACTGCCCTATCGCCATG | CATCGTGTGTGAAGAAGGTGA | AC | 28 | gi\|925159997\|ref\|NW_012341584.1\| |
| Ssa-29.d18 | 2b | AGCTACCTATTCCTGGAGCG | CCTGACCCGCTAACATCTCT | GT | 29 | gi\|925159852\|ref\|NW_012341729.1\| |
| Ssa-29.d33 | 2b | TAACTGCTGAGCCGTGTGTC | CGACGAAGATAGAATTCACTGC | AG | 29 | gi\|925159809\|ref\|NW_012341772.1\| |


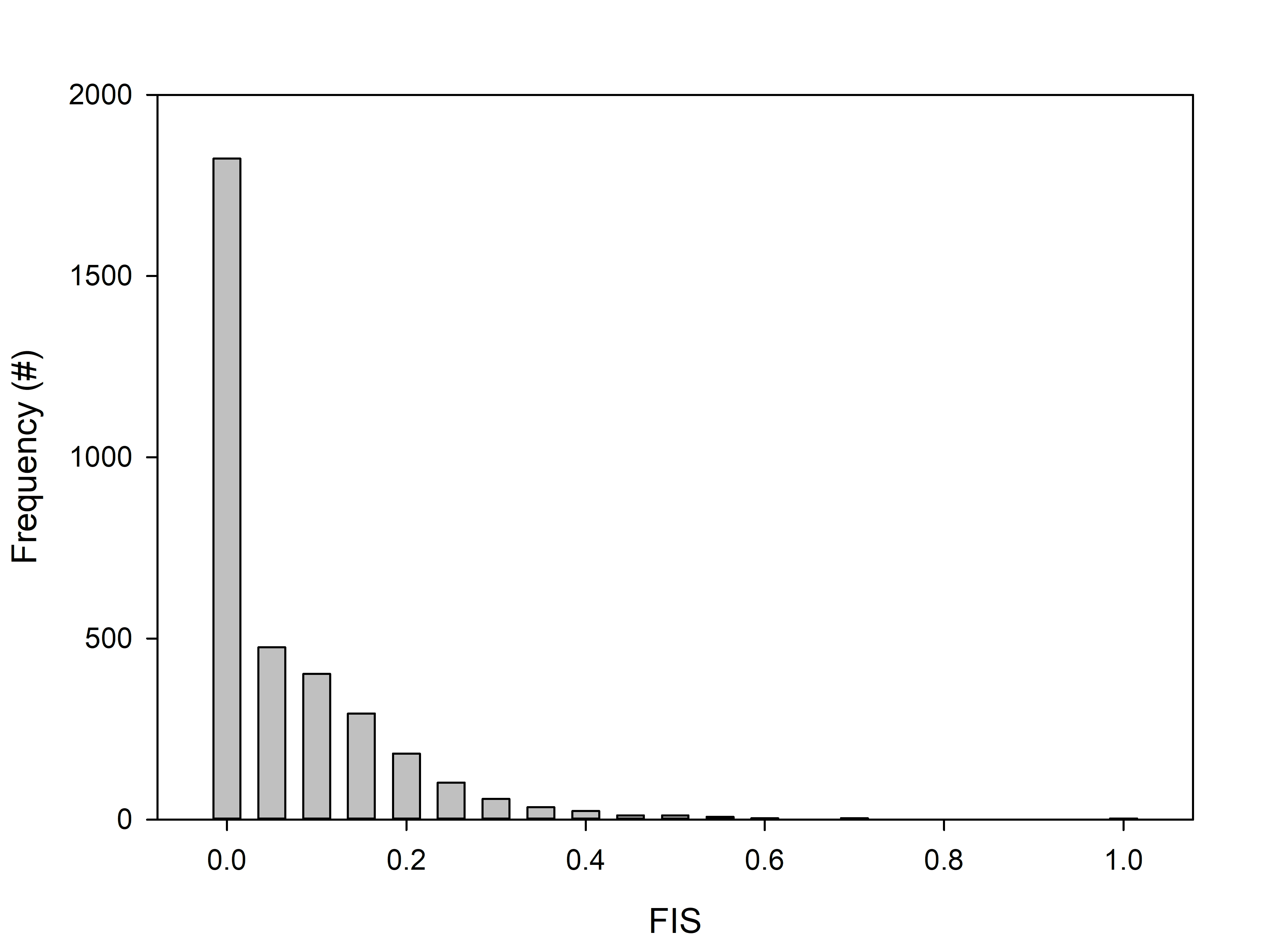


Figure S1. Frequency distribution of *F*_IS_ values for 101 microsatellite loci genotyped in 35 samples of Atlantic salmon from Labrador Canada. See Figure 1 and Table 1 for baseline sample locations.


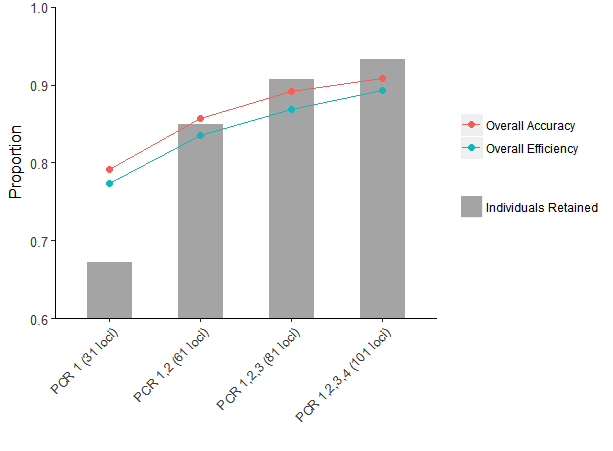


Figure S2. Overall accuracy and efficiency for incremental increases in panels by PCR multiplex. Red and blue lines represent accuracy and efficiency respectively, and grey bars represent the proportion of individuals that could be retaining at a probability threshold of 0.70.


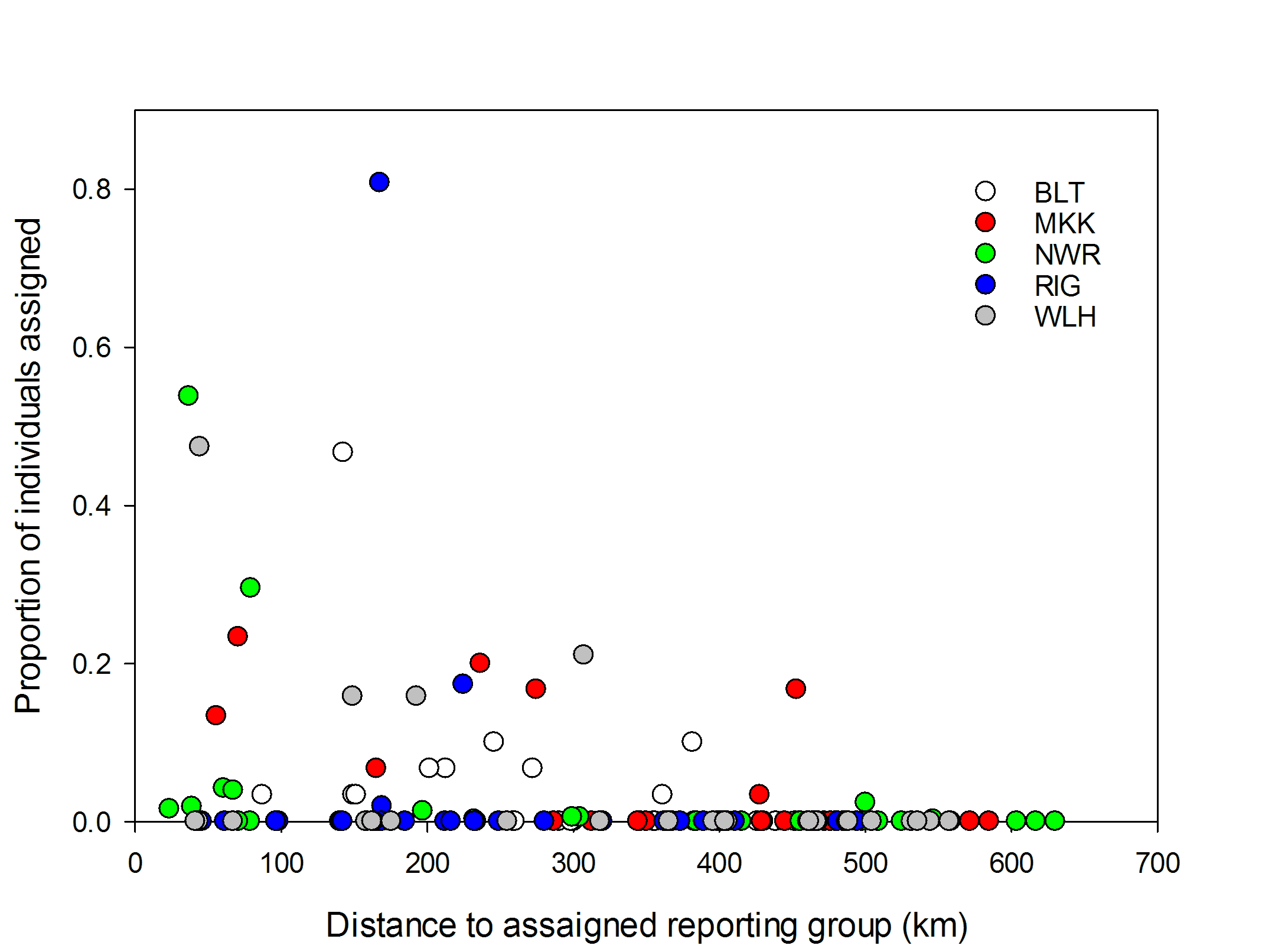


Figure S3. Individual assignments (proportion of fishery sample) with geographic distance from source reporting group for each of five fishery samples (See Methods). Circles represent fishery-specific mixture estimates. See Figure 1 and Table 1 for fishery and baseline sample locations.
